# 

**Published:** 1894-09-22

**Authors:** 


PRICE TWOPENCE.
No. 417, Vol. XVI.—Sept. 22nd, 1894.
[Registered at the General Post Office as a Newspaper for transmission in the United Kingdom and Abroad.]

WITH EXTRA NURSING SUPPLEMENT.
Per Annum, 10s. 6d., post free.
Monthly Parts, 6d.; post free, 8d.
Ditto per Annum, 8s. 6d., post free.

### THE HOSPITAL

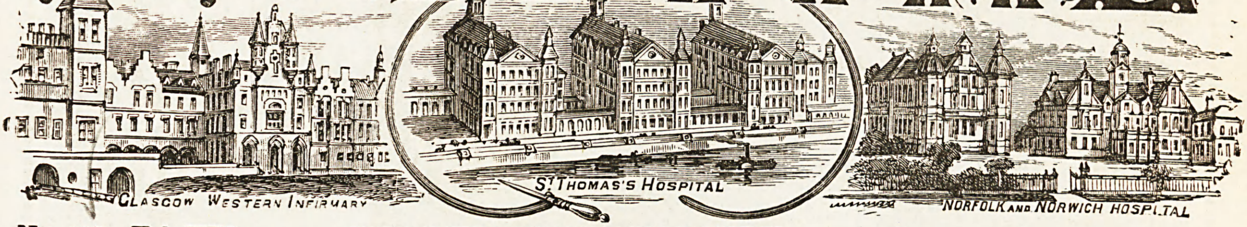

No. 417. Vol. XVI WITH EXTRA NURSING SUPPLEMENT. SATURDAY, SEPT. 22ND, 1894.

